# Facilitation of axon outgrowth via a Wnt5a-CaMKK-CaMKIα pathway during neuronal polarization

**DOI:** 10.1186/s13041-016-0189-3

**Published:** 2016-01-16

**Authors:** Shin-ichiro Horigane, Natsumi Ageta-Ishihara, Satoshi Kamijo, Hajime Fujii, Michiko Okamura, Makoto Kinoshita, Sayaka Takemoto-Kimura, Haruhiko Bito

**Affiliations:** Department of Neurochemistry, Graduate School of Medicine, The University of Tokyo, Igakubu-3-gokan, 7-3-1 Hongo, Bunkyo-ku, Tokyo 113-0033 Japan; Department of Neuroscience I, Research Institute of Environmental Medicine, Nagoya University, Nagoya, 464-8601 Japan; Department of Molecular Biology, Division of Biological Sciences, Nagoya University Graduate School of Science, Furo-cho, Chikusa, Nagoya, 464-8602 Japan; CREST, Japan Agency for Medical Research and Development, Chiyoda-ku, Tokyo 100-0004 Japan; PRESTO, Japan Science and Technology Agency, Chiyoda-ku, Tokyo 102-0076 Japan

**Keywords:** Wnt5a, Axon outgrowth, Ca^2+^ signaling, CaMKIα, CaMKK

## Abstract

**Background:**

Wnt5a, originally identified as a guidance cue for commissural axons, activates a non-canonical pathway critical for cortical axonal morphogenesis. The molecular signaling cascade underlying this event remains obscure.

**Results:**

Through Ca^2+^ imaging in acute embryonic cortical slices, we tested if radially migrating cortical excitatory neurons that already bore primitive axons were sensitive to Wnt5a. While Wnt5a only evoked brief Ca^2+^ transients in immature neurons present in the intermediate zone (IZ), Wnt5a-induced Ca^2+^ oscillations were sustained in neurons that migrated out to the cortical plate (CP). We wondered whether this early Wnt5a-Ca^2+^ signaling during neuronal polarization has a morphogenetic consequence. During transition from round to polarized shape, Wnt5a administration to immature cultured cortical neurons specifically promoted axonal, but not dendritic, outgrowth. Pharmacological and genetic inhibition of the CaMKK-CaMKIα pathway abolished Wnt5a-induced axonal elongation, and rescue of CaMKIα in CaMKIα-knockdown neurons restored Wnt5a-mediated axon outgrowth.

**Conclusions:**

This study suggests that Wnt5a activates Ca^2+^ signaling during a neuronal morphogenetic time window when axon outgrowth is critically facilitated. Furthermore, the CaMKK-CaMKIα cascade is required for the axonal growth effect of Wnt5a during neuronal polarization.

**Electronic supplementary material:**

The online version of this article (doi:10.1186/s13041-016-0189-3) contains supplementary material, which is available to authorized users.

## Background

The formation of functional neuronal circuits requires a combination of a cell’s intrinsic genetic program and extracellular factors [1]. Wnt proteins are highly conserved, secreted morphogens that activate the β-catenin-mediated canonical pathway as well as planar cell polarity and Ca^2+^-mediated non-canonical signaling pathways [2, 3]. In addition to their primary functions during the early developmental stages, Wnt proteins have been shown to regulate the cerebral cortex throughout development during various morphogenetic processes, such as anterior–posterior axis formation, neural patterning, radial migration, development of neurites (axon and dendrites), and formation of dendritic spines, both in culture and in vivo. These data indicate that Wnt signaling may play a crucial role throughout the development of the neuronal circuits [4, 5].

One of the most extensively studied Wnt proteins is Wnt5a. Wnt5a activates a non-canonical Wnt pathway that is conserved from *Caenorhabditis elegans* to humans and regulates a variety of cellular functions [6]. In the central nervous system, an unexpected role for Wnt5a and its receptor Derailed/Ryk in axon guidance has been reported in drosophila [7] and in mice [8, 9]. A gradient of Wnt5a expression has been proposed to induce the repulsion of axons in the corticospinal tract [10] and cultured neurons [9], as well as in cortical slices [11]. Paradoxically, and concurrent with this repellant activity, Wnt5a facilitated axonal outgrowth by increasing the rate of outgrowth [9]. Previous pharmacological studies have indicated that Wnt5a might activate Ca^2+^/calmodulin-dependent kinase II (CaMKII), resulting in axonal outgrowth and turning by cortical neurons [11, 12]. Furthermore, Wnt5a was also implicated in activation of PKC through a Ca^2+^ pathway that causes the axonal branching and elongation of sympathetic neurons [13]. Collectively, these studies suggested an important role for Wnt5a-activated Ca^2+^ signaling during axonal morphogenesis of several neuronal cell types.

Recent research has drawn attention to the activity of CaMKI, a distinct branch of the CaMK family, during Ca^2+^-dependent neuronal morphogenesis. CaMKI has 4 isoforms: α, β/Pnck, γ/CL3, δ/CKLiK [14–17], all of which share the requirement for both Ca^2+^/calmodulin and an upstream kinases CaMK kinase α (CaMKKα) or CaMKKβ [18–20]. Our previous studies had shown that CaMKIα facilitated axonal elongation through GABA-dependent Ca^2+^ elevation [21], while CaMKIγ promoted dendritic outgrowth through BDNF-mediated Ca^2+^ elevation [22]. Consistent with these results, inhibition of CaMKKα/β activity impaired outgrowth of both axons and dendrites in immature cortical neurons in culture [21].

Based on the above observations, we first sought to determine the time window during which Wnt5a-Ca^2+^ pathway may have a critical morphogenetic role. Combining Fluo-4 Ca^2+^ imaging and *in utero* electroporation in acute embryonic cortical slices, we tested if radially migrating cortical excitatory neurons that already bore primitive axons were sensitive to Wnt5a. While Wnt5a only evoked brief Ca^2+^ transients in immature neurons present in IZ, Wnt5a-induced Ca^2+^ oscillations were sustained in neurons that migrated out to CP. This raised the possibility that early Wnt5a-Ca^2+^ signaling during neuronal polarization has a morphogenetic consequence. Consistent with this idea, administration of Wnt5a induced axonal, but not dendritic, outgrowth in immature cortical neurons. Pharmacological and genetic inhibition of the CaMKK-CaMKI pathway abolished Wnt5a-mediated axonal elongation. Furthermore, the defective axonal growth during RNA interference against CaMKIα was rescued by a short hairpin RNA (shRNA)-resistant, wild-type CaMKIα. Collectively, our results demonstrate that CaMKK-CaMKIα is a major signaling cascade in Wnt5a-mediated axonal elongation, particularly during the early stages of neuronal polarization.

## Results

### Activation of Wnt5a-Ca^2+^ signaling in radially migrating cortical neurons

Previous findings suggested a role for Wnt5a in driving Ca^2+^ signaling during growth of callosal axons [9, 11, 12], but whether Wnt5a acted on axonal outgrowth at an earlier stage of corticogenesis was not examined. We therefore tested whether Wnt5a administration could mobilize intracellular Ca^2+^ concentrations in radially migrating cortical neurons which had just begun to extend axons in vivo. During migration, excitatory neurons transit from a multipolar to a bipolar shape at the upper IZ and exit into CP. Through a morphogenetic process that occurs in parallel to the determination neuronal cell polarity, most neurons begin to grow axons in IZ and extend them while they radially migrate into CP towards the pial surface [1, 23]. Therefore, we focused our examination of Wnt5a-Ca^2+^ signaling on bipolar-shaped neurons that had just begun to extend a primitive axon and were located in IZ and CP.

**Fig. 1:**
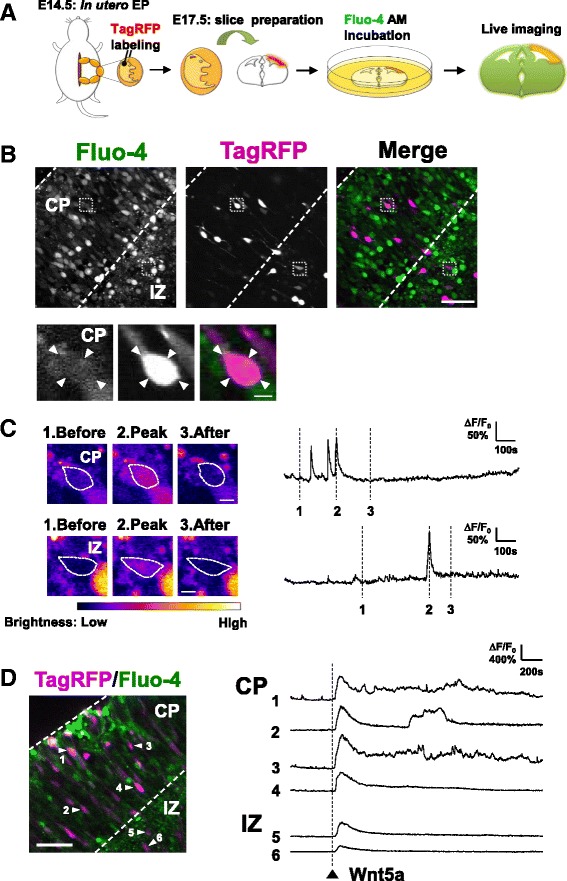
Activation of Wnt5a-Ca^2+^ signaling in radially migrating cortical neurons. **a** Protocol for Fluo-4 Ca^2+^calcium imaging in immature migrating cortical neurons. **b** Top, image acquired from a Fluo-4-labeled acute slice, in which immature migrating neurons expressing TagRFP where identified in CP and IZ. Bottom, magnified image of an immature cortical neuron in CP from the boxed region in the upper panels. Scale bars: 50 μm (top) and 10 μm (bottom). **c** Left, Fluo-4 Ca^2+^ imaging showing immature migrating cortical neurons experiencing spontaneous Ca^2+^ activity. The neurons are magnified from the boxed areas in (**b**). Right, Ca^2+^ transient traces of immature cortical neurons, with vertical lines corresponding to the time points of the left images. Scale bars: 10 μm. **d** Wnt5a administration evoked Ca^2+^ transients in immature migrating neurons, both in CP (#1–4 cells) and IZ (#5, #6 cells). Several of the immature neurons in CP (e.g. #1–3 cells) showed sustained Ca^2+^ oscillations. Scale bar: 50 μm

We transfected a cDNA encoding TagRFP, a red fluorescent protein, at E14.0 using *in utero* electroporation (IUE) to label radially migrating neurons [21, 24, 25]. Embryonic brains were prepared 65 – 70 h later, and then, Fluo-4 AM, a membrane-permeable chemical Ca^2+^ indicator, was acutely loaded into cortical slices (Fig. 1a). Migrating neurons in CP and IZ identified by TagRFP labeling (Fig. 1b) revealed spontaneous Ca^2+^ rises as visualized by transient Fluo-4 fluorescence signals (Fig. 1c, Additional file 1: Movie 1). Administration of Wnt5a onto these neurons evoked Ca^2+^ transients both in IZ and in CP. Interestingly, however, while Wnt5a-stimulated Ca^2+^ rises in IZ were brief, a sizable amount of migrating neurons in CP showed a more sustained Wnt5a-induced Ca^2+^ oscillations that lasted for at least several minutes (Fig. 1d, Additional file 2: Movie 2). These results showed that bipolar-shaped migrating neurons, which only bore primitive axons, were sensitive to Wnt5a, which could trigger a Ca^2+^ response. Furthermore, they indicated that the activation of Wnt5a-Ca^2+^ signaling might gradually develop in parallel to a morphogenetic maturation process that occurred during the transition from IZ to CP.

### Wnt5a stimulates elongation of axons but not dendrites in immature cultured cortical neurons

Our finding that Wnt5a-Ca^2+^ signaling was active in immature excitatory neurons that had just begun to extend axons in acute cortical slices was further confirmed by Ca^2+^ imaging experiments in immature cultured cortical neurons (Additional file 3: Figure S1, Additional file 4: Movie 3). These evidences prompted us to next investigate whether Wnt5a had a morphogenetic effect on immature cortical neurons during neuronal polarization.

**Fig. 2:**
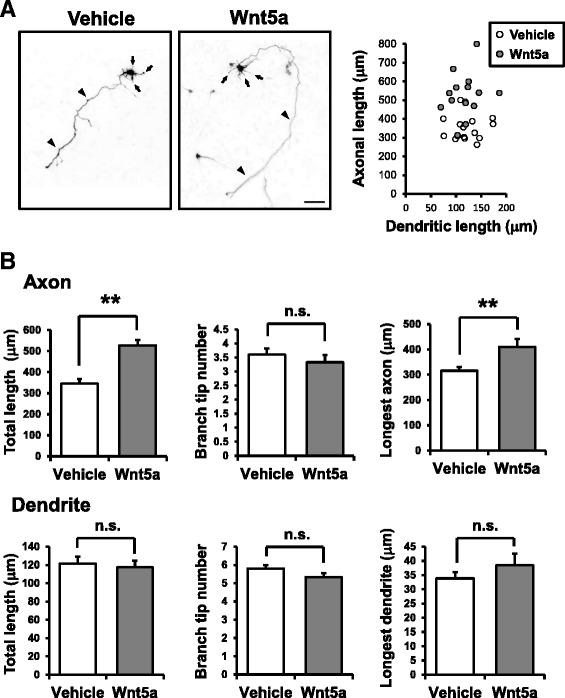
Axon-specific morphogenetic effects of Wnt5a in immature cultured cortical neurons. **a** Left, cortical neurons were stimulated with Wnt5a from 6 h until 48 h after plating. Wnt5a administration induced elongation of axons (arrowheads) but not dendrites (arrows). Right, a scatter plot of data points for both axonal and dendritic lengths obtained from individual neurons. Number of neurons: *n* = 15 for both Vehicle and Wnt5a groups; Scale bar: 50 μm. **b** Quantification of morphometric parameters in Wnt5a-stimulated neurons. For quantification, the total length, the branch tip number, and the longest processes were calculated over the axonal or the dendritic arborization for all branches exceeding 7 μm in length. Top: quantification of Wnt5a-stimulated neurons. The total length of axons and the length of the longest axons were increased. Bottom: none of the 3 morphometric parameters were significantly altered in dendrites. Number of neurons: *n* = 15 for both the vehicle and Wnt5a groups. **p < 0.01, n.s., not significant (*p* > 0.05) (t-test)

We next measured dendritic or axonal outgrowth induced by Wnt5a administration in dissociated cortical neurons during the early stages of neuronal polarization. Wnt5a was applied from 6 to 48 h after plating and morphometric analyses were then performed. Cortical neurons were blindly selected from multiple fields of view, and dendritic length (i.e., total length of all dendritic lengths) and axonal length (i.e., total length of all axonal processes, including branches) of individual cells were measured (Fig. 2a). We found that axonal outgrowth was significantly facilitated by Wnt5a administration; in contrast however, the dendritic length was not changed, demonstrating an axon-specific effect for Wnt5a during polarization. Additional quantification revealed that the Wnt5a-induced promotion of axonal outgrowth was largely attributable to the elongation of the longest axon and not an increase in the number of the branch tips (Fig. 2b). No differences were observed in the dendrites in any of these morphometric parameters. These results are consistent with the idea that Wnt5a-Ca^2+^ signaling selectively facilitated axonal elongation during neuronal polarization in the immature cortical neurons via the enhancement of primary axon outgrowth.

### Wnt5a promotes axonal elongation via a CaMKK-CaMK signaling cascade

Pharmacological studies carried out at a slightly later stages of culture have suggested that Wnt5a-Ca^2+^ signaling may be mediated by CaMKII, resulting in axon outgrowth and turning in cortical neurons [9, 11, 12]. An independent branch of Ca^2+^-dependent CaMK family comprises the CaMKI subfamily (α, β, γ, and δ), which may form several parallel kinase cascades downstream of CaMKKα and/or CaMKKβ. Recent reports, including ours, have begun to shed light on the essential role of CaMKI in the regulation of neuronal morphogenesis both in vitro and in vivo [20–22], such as promoting growth cone motility [26], neurite outgrowth [27–29], activity-dependent growth of dendrites [22, 30], and stabilization of spines [31]. Based on these findings, we critically examined whether a CaMKK-CaMK cascade may play a role in Wnt5a-Ca^2+^ signaling during early axon outgrowth.

**Fig. 3:**
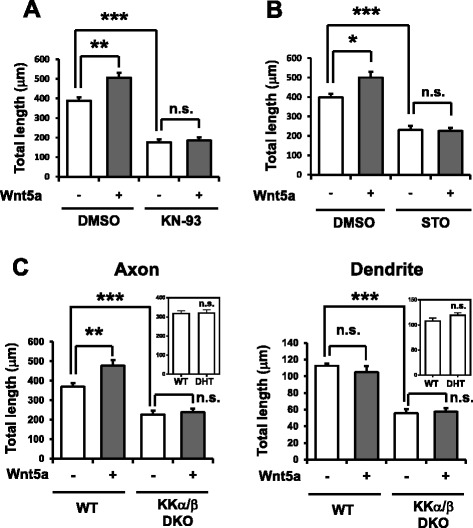
Wnt5a promotes axonal elongation via a CaMKK-CaMK cascade in immature cortical neurons. **a** Cortical neurons were stimulated with Wnt5a in the presence or absence of KN-93, a general CaMK inhibitor. Wnt5a treatment facilitated axonal elongation and this effect was potently inhibited in neurons treated with KN-93. Number of neurons: *n* = 15 for all groups. **b** Treatment with STO-609, a selective inhibitor of CaMKKα/β, the upstream kinases of CaMKI/IV, severely impaired Wnt5a-dependent facilitation of axonal elongation. Number of neurons: *n* = 15 for all groups. **c** Left, Wnt5a-dependent facilitation of axonal outgrowth was abolished in CaMKKα/β-DKO neurons. Note that the basal growth of axons was strongly impaired in CaMKKα/β-DKO neurons, in the absence of Wnt5a. Right, similar to axonal elongation, basal dendritic growth was inhibited in CaMKKα/β-DKO neurons in the absence of Wnt5a. However, dendritic growth was not facilitated by Wnt5a administration, either in the WT or DKO. Number of neurons: *n* = 15 for all groups. Insets in both graphs show no significant changes in axonal or dendritic growths in the cultured cortical neurons from either WT or CaMKKα/β double heterozygous (DHT) mice. ***p < 0.001, **p < 0.01, *p < 0.05, n.s., not significant (*p* > 0.05) (two-way ANOVA with Turkey’s test)

To test this, we first treated neurons with KN-93, which inhibits all CaMK species (CaMKII, CaMKI and CaMKIV [32–35]). We found that KN-93 potently inhibited Wnt5a-dependent axonal elongation (Fig. 3a). When neurons were exposed to STO-609, an inhibitor of CaMKK [36], Wnt5a-dependent axonal outgrowth was impaired to a similar extent as was seen with KN-93 (Fig. 3b), suggesting a possible involvement of a CaMKK-CaMK cascade. To confirm this, we generated immature cortical neurons from CaMKKα/β-double knockout (DKO), CaMKKα/β-double heterozygous (DHT), and wild type mice. Consistent with our previous studies, both axons and dendrites were significantly shortened in CaMKKα/β-DKO cortical neurons compared with cortical neurons from either WT or DHT mice (Fig. 3c). In addition to this basal phenotype, however, the axon-selective facilitation of outgrowth induced by Wnt5a was completely abolished in DKO cortical neurons (Fig. 3c). A predominant, and rather exclusive, expression of CaMKKβ in the embryonic brain, and in particular, in CP [37], and the sufficiency for a STO-609-resistant CaMKKβ mutant to rescue the STO-609-induced morphogenetic defects in immature neurons [21, 38], strongly implicate an involvement of CaMKKβ in axonogenic processes. However, a rigorous and definitive demonstration awaits combinatorial KO and rescue experiments, using STO-609-resistant forms of both CaMKKα and β [39]. Together, these results suggested that a CaMKK-CaMK cascade is required for Wnt5a-induced facilitation of axon elongation.

### An essential role of CaMKIα in mediating axon-specific facilitation of outgrowth induced by Wnt5a during neuronal polarization

**Fig. 4:**
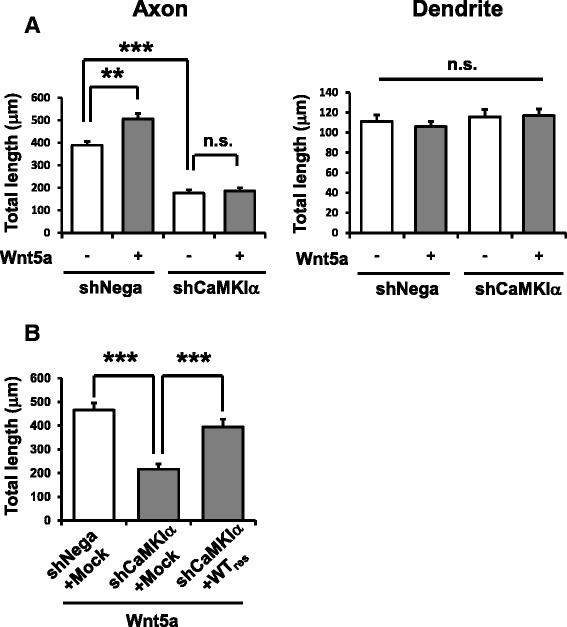
CaMKIα mediates Wnt5a-facilitated axon outgrowth. **a** Left, Specific effects of Wnt5a treatment and CaMKIα knockdown on total axonal lengths during neuronal polarization. Wnt5a treatment facilitated axon outgrowth and CaMKIα KD abolished this facilitation, in keeping with results from CaMKK blockade. Note that CaMKIα KD alone inhibited basal axonal elongation even in the absence of Wnt5a. Right, Neither Wnt5a treatment nor CaMKIα KD had any detectable effect on total dendritic lengths. Number of neurons: *n* = 15 for all groups. ***p < 0.001, **p < 0.01, n.s., not significant (*p* > 0.05) (two-way ANOVA with Turkey’s test). **b** Suppression of Wnt5a-stimulated axonal elongation by CaMKIα KD is rescued by co-expression of a shRNA-resistant wild-type CaMKIα (WTres). Number of neurons: *n* = 15 for all groups. ***p < 0.001 (one-way ANOVA with Turkey’s test)

We finally attempted to identify which kinase (likely a CaMKI/IV isoform) downstream of CaMKK mediated the facilitatory effect of Wnt5a in axonal outgrowth. The axon-specific morphogenetic effect of Wnt5a treatment was qualitatively reminiscent of results we previously reported when Muscimol, a GABA_A_ receptor agonist, was applied in immature cortical neurons: the depolarizing effect of Muscimol, by inducing Ca^2+^ elevation in immature cortical neurons, selectively promoted axonal but not dendritic outgrowth via the CaMKK-CaMKIα pathway [20, 21]. We thus evaluated whether or not CaMKIα inhibition impaired Wnt5a-dependent axon elongation. Expression of a short hairpin-type knockdown (KD) vector containing a CaMKIα-targeting sequence [21] inhibited Wnt5a-dependent axon elongation to a degree comparable to CaMKK inhibition, while dendritic length remained unaffected (Fig. 4a). Furthermore, this effect was rescued by introduction of a shRNA-resistant wild-type CaMKIα, which restored the deficit in axonal outgrowth induced by CaMKIα knockdown (Fig. 4b).

Taken together, these results provide evidence for a significant role of a CaMKK-CaMKIα cascade in mediating the facilitatory effects of Wnt5a on axon elongation during neuronal polarization. Furthermore, our findings suggest that CaMKIα may be a common axonogenic effector which mediates the effects of multiple Ca^2+^ response-inducing extracellular morphogens, such as excitatory GABA and Wnt5a.

## Discussion

In this study, we have explored a new role for Wnt5a-Ca^2+^ signaling in a very early stage of cortical morphogenesis and found that even immature migrating neurons, which just entered CP and bore only primitive axons, were already responsive to Wnt5a and capable of activating a Ca^2+^ -triggered pathway. Though the majority of studies on the in vivo effects of Wnt5a hitherto have focused on the development of commissural axons, such as those in the spinal cord and corpus callosum, our findings are consistent with a significant expression of Wnt5a mRNA in the embryonic CP [8], where immature neurons dramatically alter their shape by extending axons vertically in a direction opposite to their radial migration. While our study suggest a Ca^2+^-basis for Wnt5a-mediated control of axon growth, perhaps through facilitation of its repulsive activity [5], future studies are needed to clarify whether Wnt5a is indeed released from CP neurons and whether such ambient Wnt5a may form a gradient akin to that previously proposed in the corpus callosum surrounding commissural axons.

In keeping with our discovery of early time window of Wnt5a sensitivity in immature cortical neurons, an increase in Wnt5a signaling was recently reported to suppress canonical Wnt/β-catenin signaling, thus underlying a critical permissive role for Wnt5a during multipolar-to-bipolar morphological transition [40]. Furthermore, it has been suggested that Wnt5a proteins guide cell morphogenesis that accompanies cell migration in a variety of cellular systems [6, 41]. Thus, Wnt5a-Ca^2+^ signaling might facilitate and coordinate two cellular processes in newly bipolar shaped neurons—directional movement and axonal outgrowth, both of which are critical for proper corticogenesis.

Capitalizing on our own previous findings that a CaMKK-CaMKIα cascade might have a potent and selective axonogenic activity [21], we here have specifically investigated whether Wnt5a had a selective growth promoting effect on axons, and if so, whether a part of this might be mediated by a Ca^2+^-dependent CaMKK-CaMKIα pathway. Results from pharmacological, knockout and knockdown experiments unequivocally demonstrated a strong involvement of CaMKKs and CaMKIα in Wnt5a-faciliated axonal outgrowth. A recent study revealed that Wnt5a induced membrane insertion and clustering of functional GABA_A_ receptors that increased the amplitude of GABA currents in adult hippocampal neurons [42]. If such a mechanism were conserved in immature cortical neurons, one might imagine that direct Wnt5a effects might synergize with those of an indirect, depolarizing GABA_A_-mediated Ca^2+^ signaling in immature neurons. Alternatively, Wnt5a and GABA_A_ pathways might cooperate to evoke a common downstream Ca^2+^ signaling leading to ultimate activation of the CaMKK-CaMKIα pathway. Either way, our results indicate that CaMKIα activation would be a likely common Ca^2+^ pathway downstream of a versatile mixture of extracellular ligands. How this event then leads to phosphorylation of key substrates that modulate of cytoskeletal remodeling and membrane trafficking and trigger selective neuritic growth is currently under investigation [20, 43].

## Conclusions

This study suggests that Wnt5a activates Ca^2+^ signaling during a neuronal morphogenetic time window when axon outgrowth is critically facilitated. Furthermore, the CaMKK-CaMKIα cascade is required for the axonal growth effect of Wnt5a during neuronal polarization. These results support the notion that the CaMKIα pathway underlies axonal development in response to a wide variety of extracellular signaling molecules that are necessary to achieve precise cortical wiring.

## Methods

All recombinant DNA and animal experiments in this study were performed in accordance with regulations and guidelines for the care and use of experimental animals of the University of Tokyo and were approved by the institutional review committees of the University of the Tokyo Graduate School of Medicine.

### *In utero* electroporation

*In utero* electroporation (IUE) was performed for labeling radially migrating neurons as described previously [21, 24]. In brief, pregnant ICR mice at E14.5 or E15.5 were deeply anesthetized with Pentobarbital Sodium, and 2.0 μg/μl plasmid solution colored with Fast Green (0.1 %) was injected into the lateral ventricle of the mouse embryos through glass capillaries (GC150TF-10, Harvard Apparatus) pulled by a micropipette puller. Electroporation (5 pulses of 45 V, 50 ms) was then applied using an electroporator (ECM 830, BTX) with a tweezer-type electrode (CUY650P5, BEX Co. Ltd).

### Preparation of cortical slices

Brains were quickly removed from mice embryos 65–70 h after IUE, at E17.5, and embedded in 3 % low-melting temperature agarose (SeaPlaque Agarose, Lonza) dissolved in a solution containing: (in mM) 27 NaHCO_3_, 1.4 NaH_2_PO_4_, 2.5 KCl, 0.5 ascorbic acid, 7.0 MgSO_4_, 1.0 CaCl_2_, and 222.1 sucrose [44]. Then, each mouse brain was immersed in this ice-cold solution bubbled with a gas mixture of 95 % O_2_ and 5 % CO_2_, and 300 μm-thick coronal slices were prepared using a vibratome (VT1000, Leica). After preparation, the slices were recovered for over 30 min at room temperature in an artificial cerebrospinal fluid (aCSF) consisting of: (in mM) 127 NaCl, 26 NaHCO_3_, 1.5 KCl, 1.24 KH_2_PO_4_, 1.4 MgSO_4_, 2.4 CaCl_2_, and 10 glucose [45].

### Fluo-4 AM loading into cortical slices

In order to load Fluo-4 AM (Invitrogen), cortical slices were transferred to 35-mm dishes filled with a solution containing Fluo-4 AM (10 μM Fluo-4 AM, 0.01 % Pluronic F-127, and 0.005 % Cremophor EL were dissolved in aCSF), and incubated for 30 min at 37 °C and 5 % CO_2_ in a microincubator (Tokai Hit). After washing, the slices were maintained in a Fluo-4-free aCSF solution at room temperature for at least 30 min.

### Ca^2+^ imaging analyses

After Fluo-4 AM loading, cortical slices were transferred to a perfusion chamber (RC-22, Harvard Apparatus) attached to a LSM510META confocal laser microscopy system (Carl Zeiss) and perfused with physiological aCSF containing: (in mM) 127 NaCl, 26 NaHCO_3_, 3.3 KCl, 1.24 KH_2_PO_4_, 1.0 MgSO_4_, 1.0 CaCl_2_, and 10 glucose [44, 45] bubbled with 95 % O_2_ and 5 % CO_2_ at 37C°. Time-lapse Fluo-4 and TagRFP images were acquired sequentially using an oil-immersion objective (Plan-Neofluar 40×/1.30NA, Carl Zeiss) and Multi-track mode of data collection software (Zen, Carl Zeiss) at 0.5 fps with 512 × 512 pixels. Fluo-4 and TagRFP were excited by Argon (488 nm) and HeNe (543 nm) laser lines, and their emission signals were selected by 505-550 band-pass filter and 561-615 spectral imaging detector, respectively. After baseline imaging, aCSF containing 10× Wnt5a (1× final concentration: 100 nM) was gently bath-applied. Fluorescence signals in the cell bodies of individual migrating neurons, identified by TagRFP expression, were quantified using Image J software.

For quantification of Fluo-4 intensity, region of interest (ROI) was extracted from each frame based on TagRFP images. After background subtraction, the change in Fluo-4 fluorescence intensity was defined as:

$$ \Delta {\mathrm{F}}_{\left(\mathrm{Fluo}\hbox{-} 4\right)}/{\mathrm{F}}_{0\left(\mathrm{Fluo}\hbox{-} 4\right)} = \left({\mathrm{F}}_{\left(\mathrm{Fluo}\hbox{-} 4\right)}\hbox{--}\ {\mathrm{F}}_{0\left(\mathrm{Fluo}\hbox{-} 4\right)}\right)/{\mathrm{F}}_{0\left(\mathrm{Fluo}\hbox{-} 4\right)} $$

where F_0(Fluo-4)_ is the averaged intensity of baseline; for detection of spontaneous Ca^2+^ activity, baseline was calculated based on the fluorescence intensity of bottom 75 % ranked frames; for imaging of Wnt5a-evoked Ca^2+^ responses, baseline was calculated based on the fluorescence intensity of all frames preceding Wnt5a application); and F_(Fluo-4)_ is the fluorescence intensity measured at the given time points.

To correct for motion and focusing artifacts during the imaging of migrating neurons, we normalized Fluo-4 fluorescence intensity against the fluorescence of the volume-filling TagRFP. Briefly, we calculated F_(TagRFP)_/F_0(TagRFP)_ from the fluorescent intensity of TagRFP after background subtraction, where F_0(TagRFP)_ is the averaged intensity of all imaging periods, and F_(TagRFP)_ is the fluorescence intensity at any time point. Finally, ΔF/F_0_, expressed as a percentage, was calculated as (ΔF_(Fluo ‐ 4)_/F_0(Fluo ‐ 4)_)/ (F_(TagRFP)_/F_0(TagRFP)_).

For Ca^2+^ imaging of immature cultured cortical neurons, a G-CaMP7-based Ca^2+^ indicator [46] and S.K. et al. in preparation was expressed in cortical layer 2/3 neurons by IUE of ICR mice at E15.5, and expressing hemispheres were collected at E18.5. Following mild trypsin digestion and gentle mechanical trituration, 2.0 × 10^5^ dissociated neurons were plated onto poly-D-lysine coated glass bottom dishes (MatTek) and maintained for two days in minimal essential medium (MEM) supplemented with 10 % fetal bovine serum (FBS) and with 2 % NS21 [47]. All imaging experiments were performed at 37 C° and 5 % CO_2_ in a stage top incubator (Tokai Hit) mounted on an IX71 microscope (Olympus). The images were acquired at 256 × 256 pixels using an oil-immersion lens (60×/1.49NA) and a C9100-12 EM-CCD (Hamamatsu Photonics) at 1fps with an exposure time of 100 ms. We used FF01-474/23, FF02-520/28 filters (Semrock) as excitation and emission filters respectively. The excitation light was supplied by a Lambda-DG4 (Sutter Instruments) Xenon lamp light source. During imaging, 5 × concentrated Wnt5a-containing culture medium (500 ng/ml) was manually added to the imaging dish with a syringe.

For quantification of G-CaMP fluorescence, regions of interest (ROIs) were obtained from the first frame of time-lapse images. After background subtraction, the change in G-CaMP fluorescence intensity was defined as:

$$ \Delta \mathrm{F}/{\mathrm{F}}_0 = \left(\mathrm{F}\hbox{--}\ {\mathrm{F}}_0\right)/{\mathrm{F}}_0 $$

where F is the fluorescence intensity at given time points and F_0_ is the averaged intensity of all frames preceding Wnt5a application.

### Preparation of primary cortical cultures for morphometric analysis

Primary cultures of immature cortical neurons were prepared from embryonic day 19 (E19) Sprague-Dawley rats or E17 C57BL/6 mice (wild-type and DKO mutant mice) were prepared as previously described [21]. In brief, after dissection, cortices were incubated for 10 min with 10 mg/ml trypsin type XI (Sigma-Aldrich) plus 0.5 mg/ml DNase I type IV (Sigma-Aldrich) at room temperature and mechanically dissociated in Hanks’ solution, pH 7.4 (Sigma-Aldrich), with 0.5 mg/ml DNase I type IV and 12 mM MgSO_4_. Dissociated neurons were transfected by electroporation using Nucleofector (Amaxa Biosystems) and plated onto either poly-L-lysine-coated 12 mm coverslips (BD Biosciences), poly-D-lysine-coated glass-bottom dishes (MatTek), or six-well dishes (BD Biosciences), and cultivated in minimum essential medium (Invitrogen) containing 5 g/L glucose, 0.2 g/L NaHCO_3_, 0.1 g/L transferrin (Calbiochem), 2 mM GlutaMAX-I (Invitrogen), 25 μg/ml insulin (Sigma-Aldrich), B-27 supplement (Invitrogen), and 10 % fetal bovine serum. All primary cortical cultures were incubated in 5 % CO_2_ at 37 °C.

### Pharmacological stimulation and inhibition experiments

Wnt5a (R&D Systems), KN-93 (Calbiochem), or STO-609 (Tocris Bioscience) were applied to the medium of cultured cortical neurons expressing mRFP1 for morphometric analysis from 6 h after plating onwards [21]. Final drug concentrations were 400 ng/ml (Wnt5a), 10 μM (KN-93), or 2.6 μM (STO-609). Bath application was performed by dissolving the reagents in one-half volume of the conditioned culture medium and then mixing this volume gently with the remaining volume of the original medium that remained in the dish. The culture medium was not changed before fixation.

### Construction of shCaMKIα vector and shRNA-resistant wild-type CaMKIα vector

For RNAi experiments, a shCaMKIα vector co-expressing mRFP1 as a morphological tracer was constructed essentially as described [21]. In brief, to create pSUPER-shCaMKIα complementary 60-bp oligonucleotides carrying antisense and sense sequences for CATTGTAGCCCTGGATGAC (19-bp, corresponding to nucleotides 231–249 of mouse CaMKIα) were subcloned into the pSuper + mRFP1 plasmid backbone. pSUPER-shNega was generated similarly, except that an artificial 19-mer sequence (ATCCGCGCGATAGTACGTA) was used as a target [21]. This sequence was based on a commercially available negative control siRNA sequence (B-Bridge International) that we confirmed had no significant similarity to any known mammalian gene using the Basic Local Alignment Search Tool (BLAST). For generating the shCaMKIα-resistant construct, silencing mutations were introduced into the shCaMKIα target sequence of rat wildtype CaMKIα cDNAs [16], and the mutated rat CaMKIα cDNA was inserted into the pEGFPC1 vector (BD Clontech).

### Generation of the CaMKKα/β-DKO mouse line

CaMKKα-KO mice have been described previously [48], and CaMKKβ-KO mice were previously created [21, 49]. In brief, similar to CaMKKα-KO mice, exon 2 (harboring the ATG start codon) through exon 6 of the CaMKKβ gene was deleted in CaMKKβ-KO mice. A detailed characterization of the CaMKKβ-KO mice has been published [49]. CaMKKα-KO and CaMKKβ-KO mice were crossed to produce a CaMKKα/β-double knockout (DKO) mouse line. The DKO line and wild type C57BL/6 mice were crossed to produce a CaMKKα/β-double heterozygous (DHT) mouse line as a control.

### Quantitative analysis of axons and dendrites

For quantification of process length and tip number, cortical cultures were transfected with plasmids encoding CAG promoter-driven mRFP1 by electroporation, plated onto 12-mm poly-L-Lysine-coated coverslips at the density of 5 × 10^5^ cells (rats) or 7.5 × 10^5^ cells (mice) per coverslip in 24-well plates, and then fixed at 2 Days In Vitro. Morphometric analyses were performed using immunofluorescence images of mRFP1 captured by an Olympus BX51 microscope system with a 20 × objective. Axons and dendrites were identified using standard morphological criteria and only neurons that exhibited one clearly classifiable axon and one or more dendrites were analyzed. For all quantitative analyses, the observer was blinded to the identity of the transfected constructs, mice genotypes, and drug treatments.

### Statistical analyses

Statistical analyses were performed using Prism 4.0 (GraphPad Software). The student’s t-test was used for comparisons of the two groups. One- or two-way analysis of variance (ANOVA) with *post-hoc* Turkey-Kramer or Bonferroni test was used for factorial analysis between three or more groups. All data are reported as the mean ± standard error of mean (SEM).

## Additional files

Additional file 1: Movie 1. Fluo-4 Ca^2+^ imaging of immature migrating cortical neurons during spontaneous Ca^2+^ activity. Movie 1 (related to Fig. 1c) shows transient Fluo-4 fluorescence signals induced by spontaneous Ca^2+^ rises, in migrating neurons in CP and IZ as identified by TagRFP expression. Migrating neurons in CP and IZ are magnified from boxed areas in the left panel of the movie. (AVI 3964 kb) Additional file 2: Movie 2. Wnt5a-evoked Ca^2+^ transients in immature migrating neurons, both in CP and IZ. Movie 2 (related to Fig. 1d) shows a robust Wnt5a-evoked Ca^2+^ response in the acute cortical slice. Appearance of a white square in the upper left corner indicates the time window of Wnt5a application. Several of the immature neurons in CP (e.g. #1–#3 cells) showed sustained Ca^2+^ oscillations. (AVI 4670 kb) Additional file 3: Figure S1. Activation of Wnt5a-Ca^2+^ signaling in immature cultured cortical neurons. Addition of Wnt5a (100 ng/ml, final concentration) to the culture medium evoked a sustained Ca^2+^ oscillatory response in immature cultured cortical neurons (Cell #1 and Cell #2). Vertical lines indicate the time points of Wnt5a application and of the respective image frames. Scale bar: 5 μm. (PDF 74 kb) Additional file 4: Movie 3. Wnt5a-induced sustained Ca^2+^ oscillations in an immature cultured cortical neuron. Movie 3 (related to Additional file 1: Figure S1) shows a sustained Wnt5a-induced Ca^2+^ oscillatory response in a primary cortical culture at 2DIV. Appearance of a white square in the upper left corner indicates the time window of Wnt5a application. (AVI 5522 kb)
