# Supplementary figures and images for: Facilitation of axon outgrowth via a Wnt5a-CaMKK-CaMKIα pathway during neuronal polarization
Source: Mol Brain. 2016 Jan 16;9:8. doi: 10.1186/s13041-016-0189-3 (PMC4715351; doi:10.1186/s13041-016-0189-3)

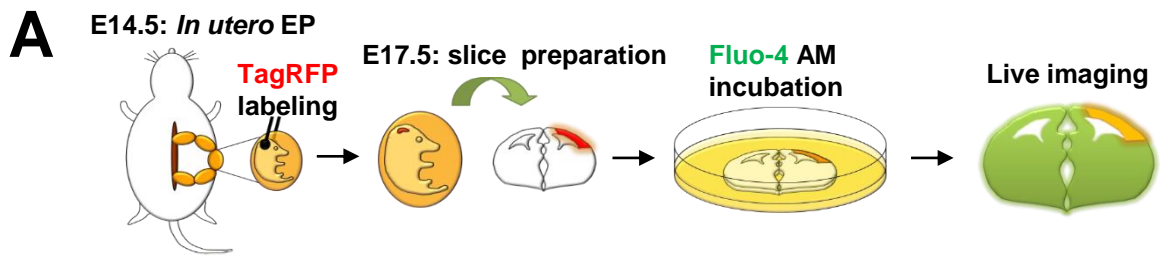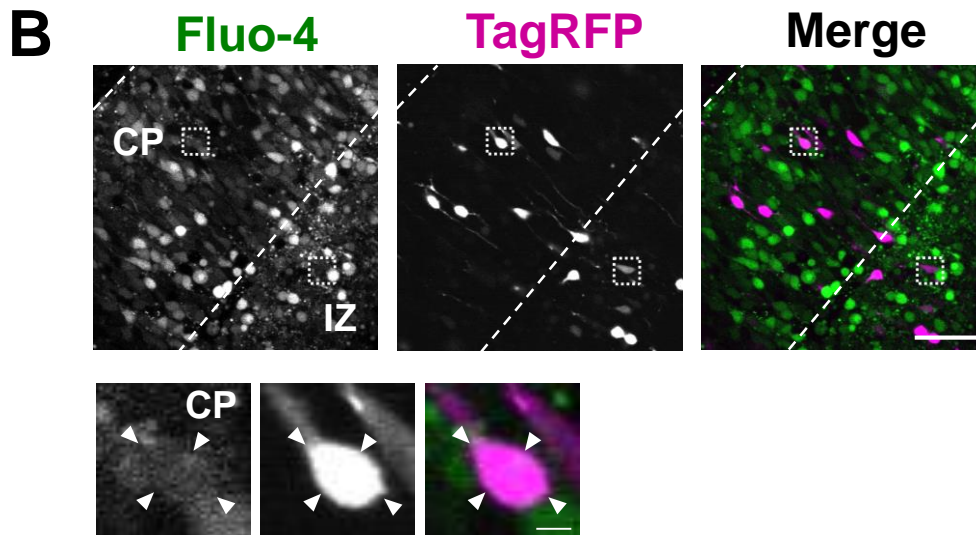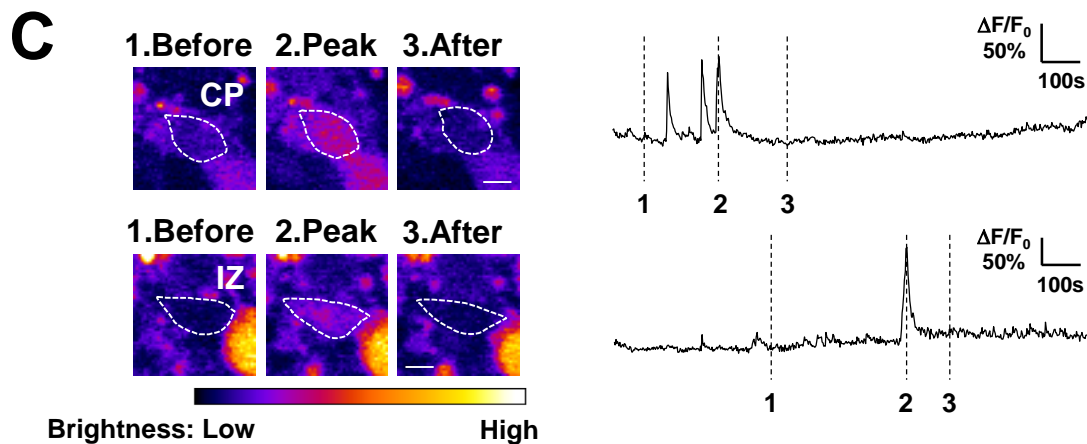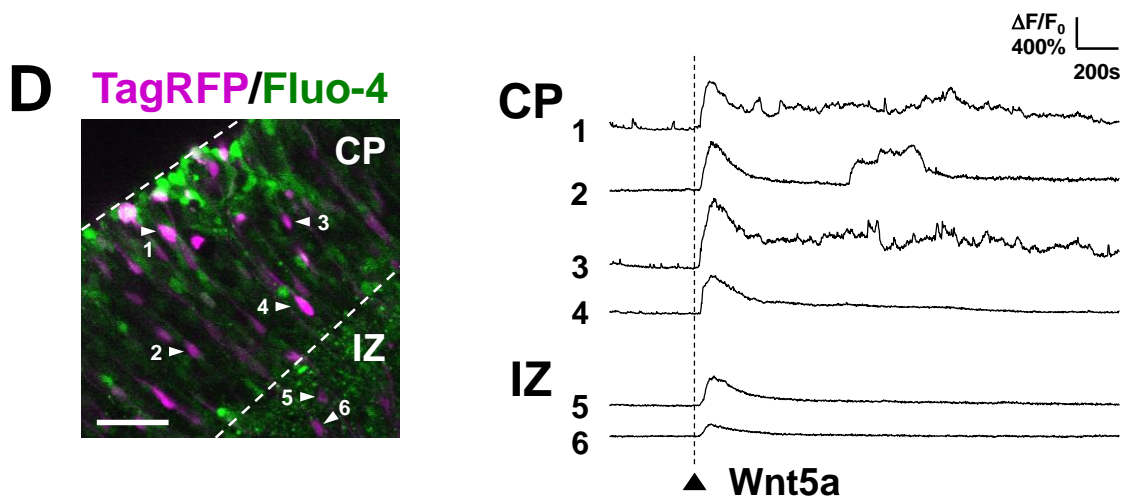

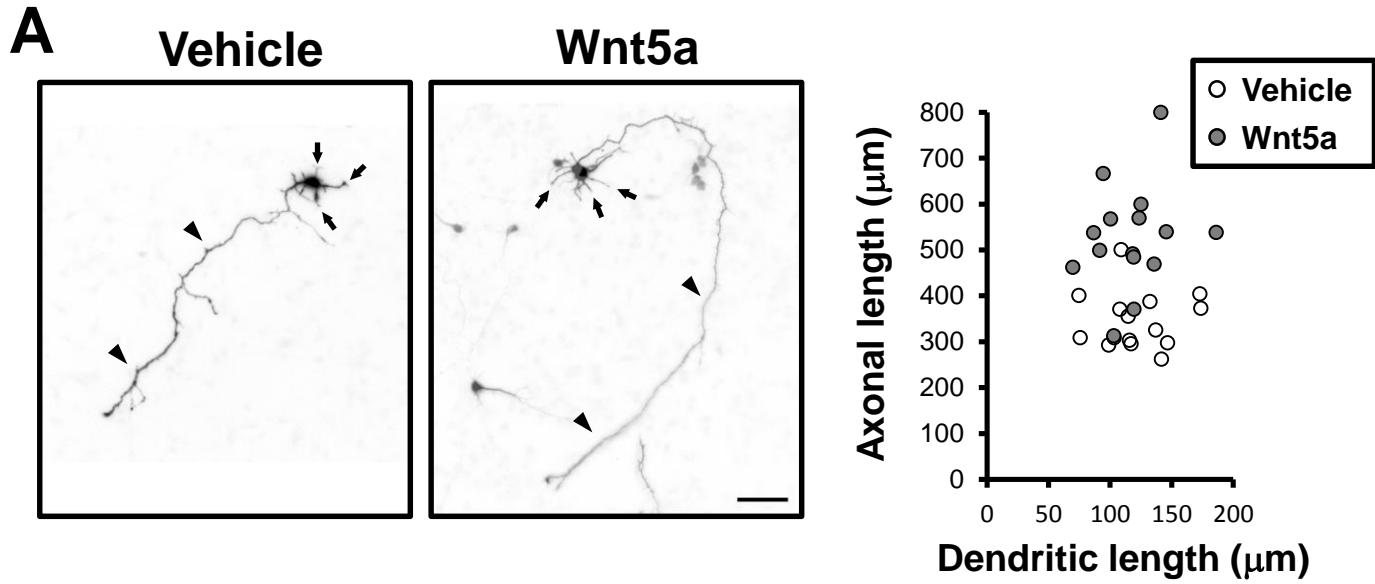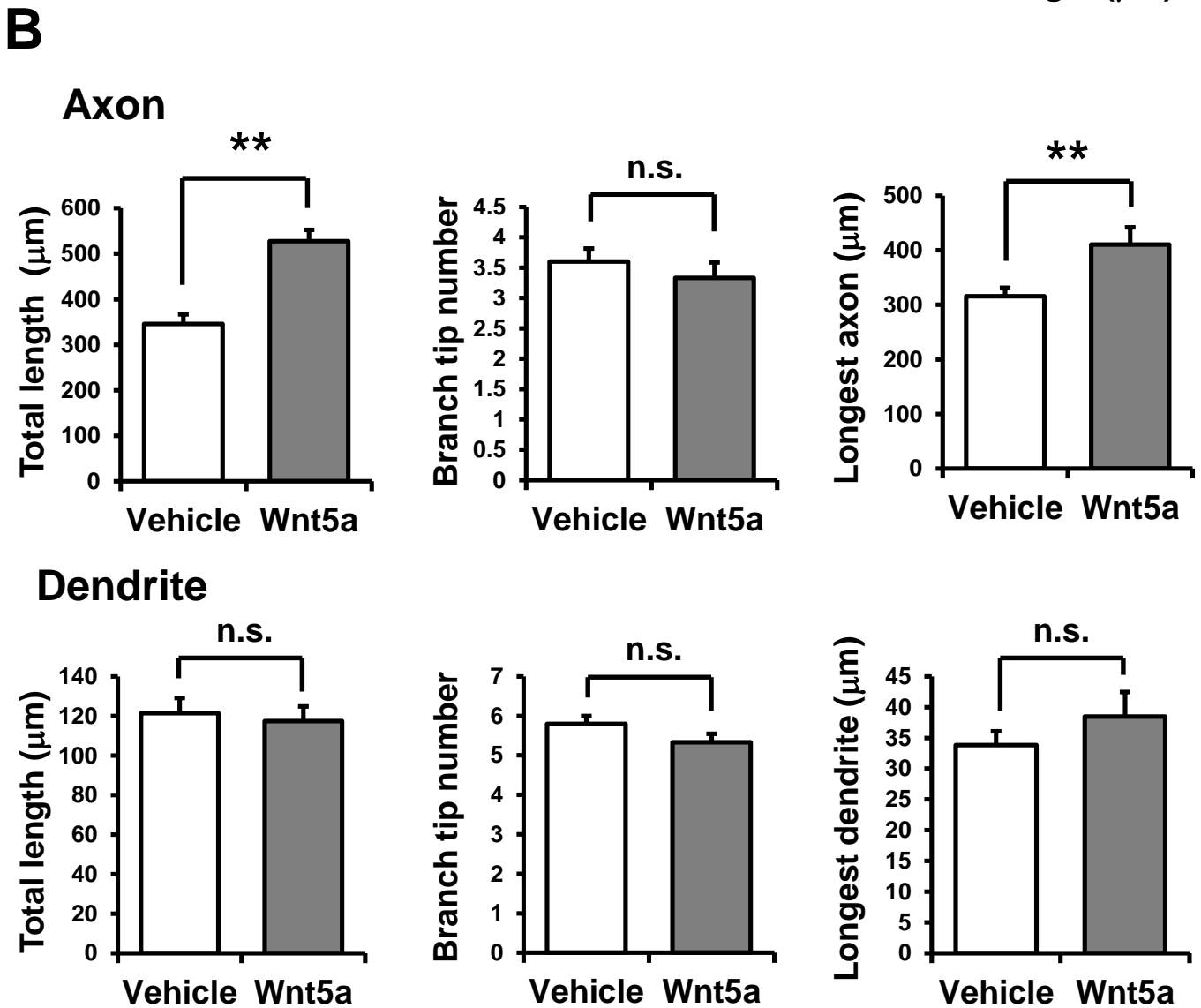

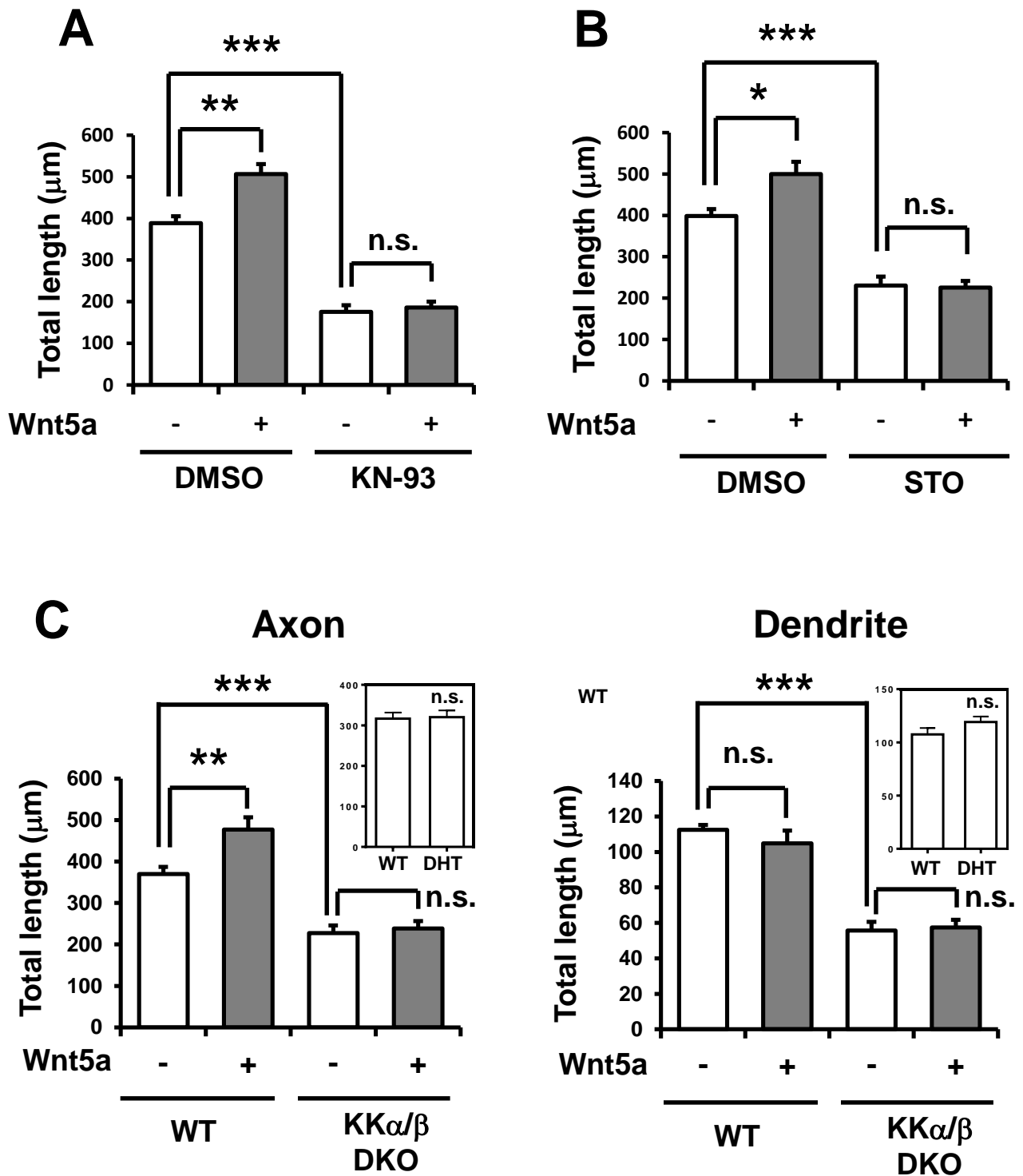

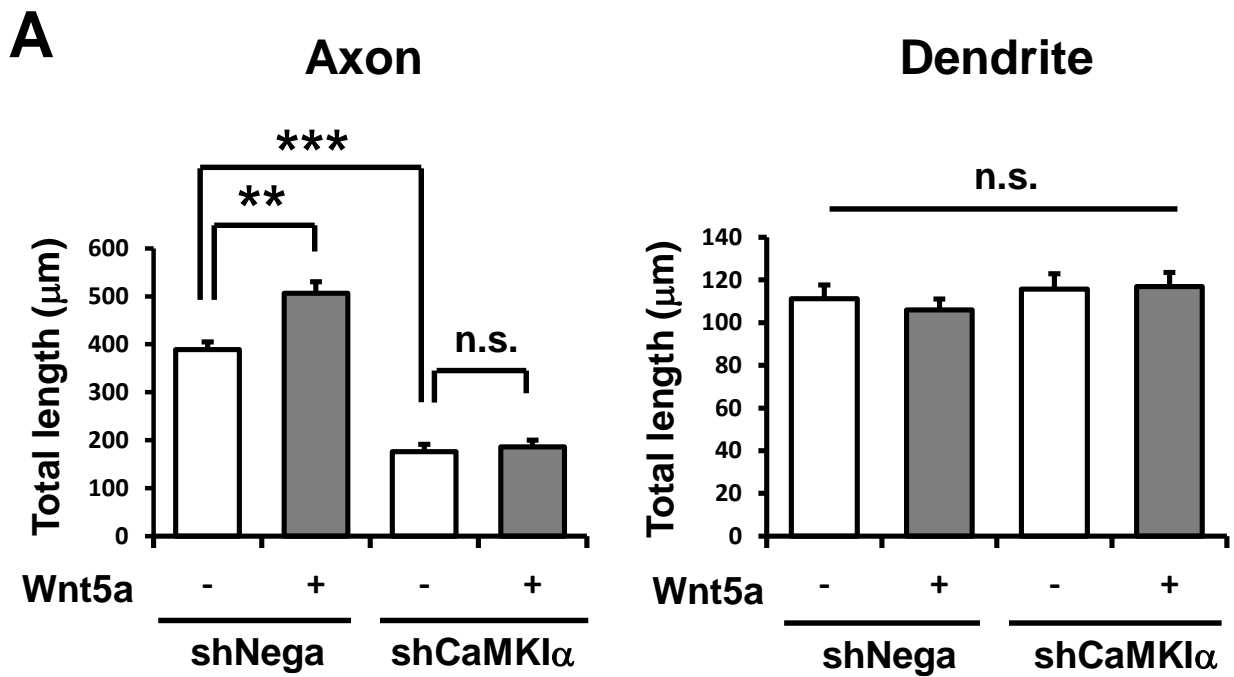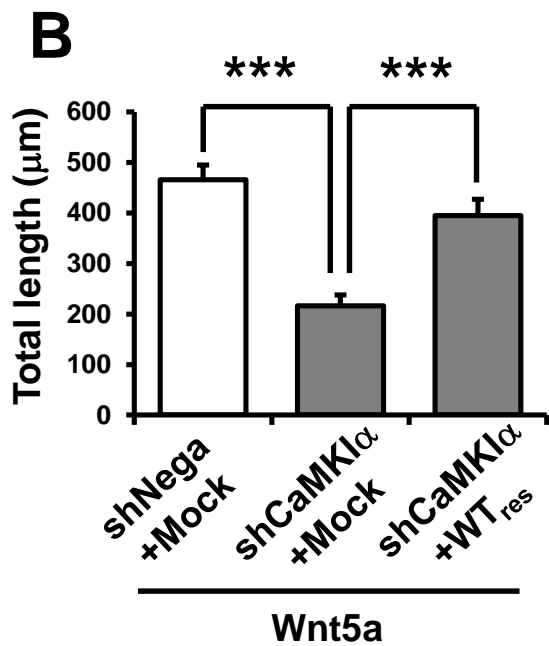

Supplemental Figure 1 Horigane et al.

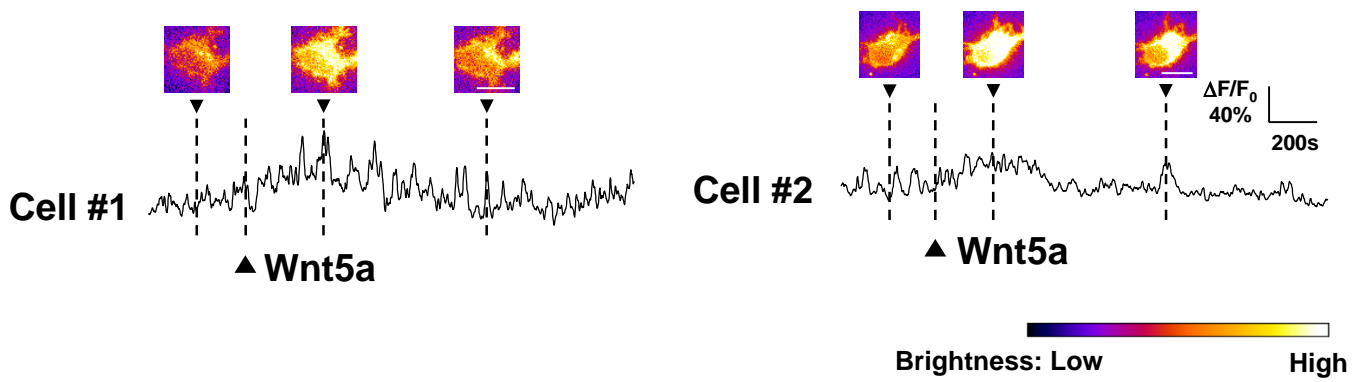

Supplement: Additional file 3: Figure S1. — Activation of Wnt5a-Ca2+ signaling in immature cultured cortical neurons. Addition of Wnt5a (100 ng/ml, final concentration) to the culture medium evoked a sustained Ca2+ oscillatory response in immature cultured cortical neurons (Cell #1 and Cell #2). Vertical lines indicate the time points of Wnt5a application and of the respective image frames. Scale bar: 5 μm. (PDF 74 kb) [file 13041_2016_189_MOESM3_ESM.pdf]
